# Increasing genetic diversity of Zika virus in the Latin American outbreak

**DOI:** 10.1038/emi.2016.68

**Published:** 2016-07-06

**Authors:** Weifeng Shi, Zhenjie Zhang, Cheng Ling, Michael J Carr, Yigang Tong, George F Gao

**Affiliations:** 1 Institute of Pathogen Biology, Taishan Medical College, Taian, Shandong Province 271000, China; 2 College of Information Science and Technology, Beijing University of Chemical Technology, Beijing 100029, China; 3 Global Station for Zoonosis Control, Global Institution for Collaborative Research and Education (GI-CoRE), Hokkaido University, Sapporo 001-0020, Japan; 4 National Virus Reference Laboratory, University College Dublin, Dublin 4, Ireland; 5 State Key Laboratory of Pathogen and Biosecurity, Beijing Institute of Microbiology and Epidemiology, Beijing 100101, China; 6 Chinese Center for Disease Control and Prevention, Beijing 102206, China; 7 Institute of Microbiology, Chinese Academy of Sciences, Beijing 100101, China

**Dear Editor,**

The spread of the emerging zoonotic arboviral agent, Zika virus (ZIKV), in the Americas and its association with congenital abnormalities,^1, 2^ including *in utero* growth restriction, placental insufficiency, microcephaly and fetal death, as well as neurological conditions, such as Guillain Barré syndrome, has led the World Health Organization to declare a public health emergency of international concern on 1 February 2016. As of 17 March 2016, 33 countries and territories in Latin America and the Caribbean have reported autochthonous transmission of ZIKV.^3^ In addition, a further 20 countries and territories have reported imported ZIKV infections in Asia (China), Europe (France, Netherlands and Spain)^4^ and North America (United States of America and Canada), which has raised concerns of the potential for large-scale outbreaks in equatorial regions and the northern hemisphere, where ~80% of the human population reside.

So far, at least two hypotheses have been proposed to account for the unexpectedly large outbreak of ZIKV in Latin America. First, the exceptional climatic conditions, arising from the strong El Niño event in 2015, in northeastern South America may have contributed to the rapid dispersal of ZIKV.^5^ Second, virological factors might also be associated with the rapidly expanding ZIKV epidemic, such as the putative recombination event in the *NS2B* region between ZIKV and Spondweni virus.^6^ However, it remains poorly understood why sporadic ZIKV infections were identified prior to 2013 and large-scale outbreaks have occurred since 2014.

In the present study, all available full-length ZIKV genome sequences were downloaded from GenBank on 28 March 2016. For sequences with an isolation year of 1947, a single representative MR 766 prototype (GenBank accession no. AY632535) was kept and the others were removed from the analysis. Finally, our data set included 56 complete ZIKV genome sequences, including 34 from the ongoing Latin American outbreak since 2015. Multiple sequence alignment was performed using Muscle.^7^ Phylogenetic analysis was performed using three different methods. The maximum likelihood analysis was performed using RAxML,^8^ with the GTRGAMMA model applied and 1000 bootstrap replicates. Phylogenetic analysis and demographic reconstruction were jointly estimated using Bayesian Evolutionary Analysis by Sampling Trees v1.8,^9^ using the strict and uncorrelated lognormal relaxed models, respectively. For tree priors, the Gaussian Markov random field Bayesian skyride model was used. Fifty million steps were run, and the first 10% were removed as burn-in.

A recent phylogenetic analysis based on viral envelope gene sequences has classified ZIKV into two major genetic lineages, African and Asian, and the Latin American ZIKV outbreaks segregate with the Asian lineage.^10^ Our results supported the Asian lineage origins of the currently circulating ZIKV (Figures 1A and 1B;Supplementary Figure S1). Furthermore, phylogenetic analysis revealed that the Asian lineage has evolved into two major lineages with high statistical support (Figures 1A and 1B), which we term the Oceanian and Latin American lineages. The Oceanian lineage included three imported Chinese ZIKV cases, all of whom returned from Fiji (Melanesia) and Samoa (Polynesia). This revealed an independent ZIKV lineage currently circulating in countries in Oceania.

Consistent with a previous report,^11^ our results also supported a single introduction event of ZIKV to Latin America (Figures 1A and 1B), and the estimated time of this event was dated around mid-2013 (Figure 1B). This was in agreement with a previous analysis of the time to the most recent common ancestor of all Brazilian genomes.^11^ Soon after being imported to Latin America, ZIKV became highly diversified between late 2013 and early 2014 (Figure 1B), which was supported by the co-existence of several minor clusters, although statistical support for some of these clusters was not very high (Figures 1A and 1B). These results implied that the ZIKV responsible for the current outbreak in Latin America may have become phylogenetically diversified and increased in genetic diversity.

The Brazilian ZIKV genomes did not cluster together rather, they were interspersed among the trees clustering with genomes' sampled worldwide isolates, indicative of a high level of genetic diversity of ZIKV in Brazil, although whether this arose from multiple introductions to Brazil remains unknown, based on current data.^11^ In addition, in the Latin American lineage, ZIKV genomes collected from 2015 and 2016 did not group together and were distributed throughout the clusters with no evidence of substantial lineage replacement (Figures 1A and 1B). This suggests that no circulating ZIKV strain has gained significantly higher fitness over the others to become dominant.

The evidence for increasing genetic diversity was also supported by the demographic reconstruction analysis (Figure 1C). Since late 2013, when the ZIKV that established the current epidemic in Latin America was introduced, the genetic diversity of ZIKV has gradually increased and reached its peak in approximately March or April 2015, following a plateau period until September 2015. During this period of time, the Latin American lineage became diversified and co-circulated with the Oceanian lineage. Although the analysis, based on the currently available data, suggested that the genetic diversity decreased slightly after September 2015 (Figure 1C), we consider that this is potentially caused by the extremely low sampling density in Latin America.

In summary, we present phylogenetic evidence of the co-circulation of two major ZIKV lineages in Oceania and Latin America. In addition, the Latin American lineage has become highly diversified. The genetic diversity of ZIKV may have been gradually increasing since late 2013 with its geographic expansion, reaching a peak in March or April 2015. Based on current evidence, no circulating strain of the ZIKV Latin American lineage has become dominant. However, it is extremely likely that the genetic diversity of ZIKV is underestimated due to the limited sequence data that are currently available for Latin America and also importantly for Oceania (Polynesia, Melanesia and Micronesia) and Southeast Asia. Improved sampling efforts in vector species and human cases from these regions will help to better elucidate the evolution of this zoonotic pathogen, assist efforts to validate robust serological and molecular diagnostic assays, and identify stable epitopes for vaccine development.

## Figures and Tables

**Figure 1:**
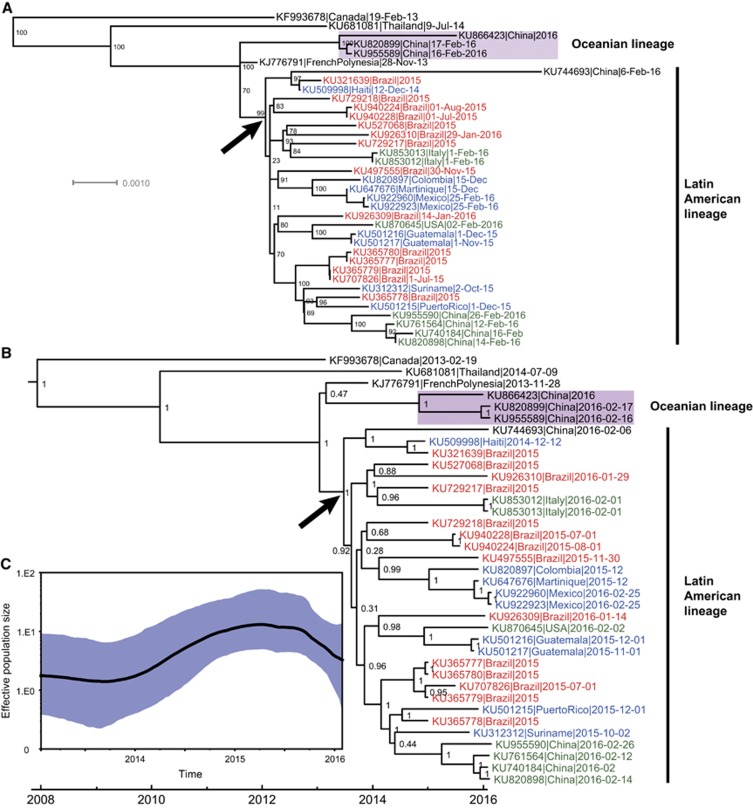
Phylogenetic analysis and demographic reconstruction of ZIKV genome sequences. Phylogenetic trees of full-length ZIKV genome sequences from the Asian lineage since 2013 (**A**: maximum likelihood; **B**: BEAST using a strict molecular clock). In both (**A**) and (**B**), viruses from Brazil are labeled in red, those from other Latin American/Caribbean countries in blue, and those from outside Latin America in green. The arrows in both panels represent the introduction event of ZIKV to Latin America. (**C**) The demographic reconstruction of the complete ZIKV genome sequences of the Asian lineage since 2013. This was estimated using the uncorrelated lognormal relaxed molecular clock and the GMRF Bayesian skyride model. Abbreviations: Bayesian Evolutionary Analysis by Sampling Trees, BEAST; Gaussian Markov random field, GMRF; Zika virus, ZIKV.
