# Supplementary material for: Increasing genetic diversity of Zika virus in the Latin American outbreak
Source: Emerg Microbes Infect. 2016 Jul 6;5(7):e68–. doi: 10.1038/emi.2016.68 (PMC4972906; doi:10.1038/emi.2016.68)
Supplement: Supplementary Figure S1 [file emi201668x1.pdf]

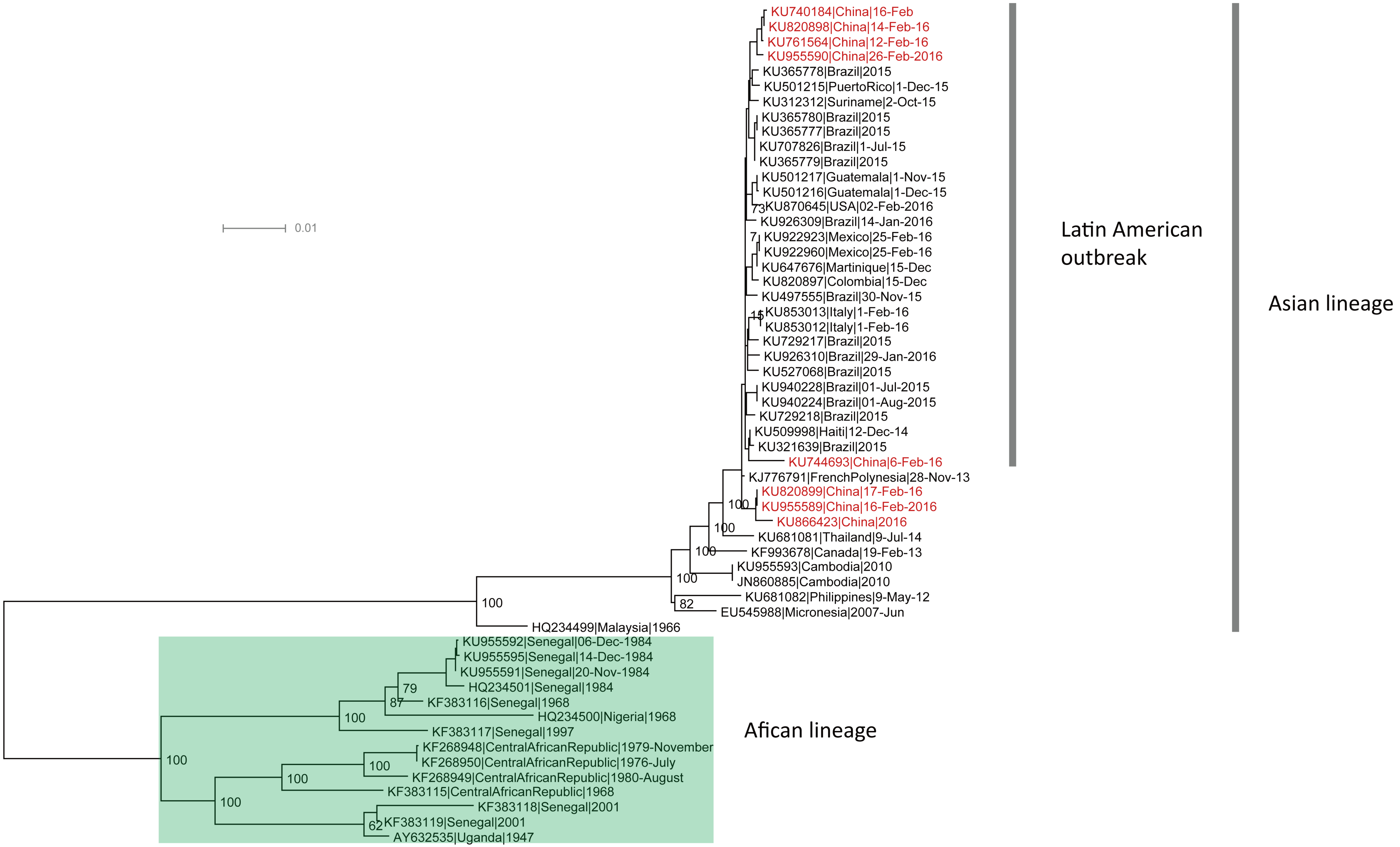

Supplementary Figure S1. The maximum likelihood phylogeny of 56 full-length ZIKV genome sequences.
